# 45698 Molecular Signatures of Cocaine Neurotoxicity in Human Brain Models

**DOI:** 10.1017/cts.2021.693

**Published:** 2021-03-30

**Authors:** Emily Mendez, Laura Stertz, Gabriel Fries, Ruifeng Hu, Thomas Meyer, Zhongming Zhao, Consuelo Walss-Bass

**Affiliations:** University of Texas Health Science Center at Houston

## Abstract

ABSTRACT IMPACT: This project will use human neuron models and bioinformatics techniques to elucidate mechanisms of cocaine neurotoxicity, which will allow treatments to be developed for minimizing or preventing neurological damage caused by cocaine abuse and overdose. OBJECTIVES/GOALS: The goals of this project are to identify genes and gene networks altered by cocaine exposure in neurons (short term), and to use these pathways to understand mechanisms of cocaine neurotoxicity for the establishment of therapeutic targets (long term). METHODS/STUDY POPULATION: To study the molecular effects of cocaine, we generated preliminary proteomics and next-generation RNA sequencing (RNAseq) data from human postmortem prefrontal cortex (Broadmann area 9 or BA9) of 12 cocaine overdose subjects and 17 controls. Future directions for this project include RNAseq analysis of neuronal nuclei sorted from human postmortem BA9 and a human induced pluripotent stem cell-derived neuron (hiPSN) model of cocaine exposure from the same postmortem subjects from whom we have brain samples. RESULTS/ANTICIPATED RESULTS: We found alterations in neuronal synaptic protein levels and gene expression, including the serotonin transporter SLC6A4, and synaptic proteins SNAP25, SYN2, SYNGR3. Pathway analysis of our results revealed alterations in specific pathways involved with neuronal function including voltage-gated calcium channels, and GABA receptor signaling. In the future, we expect to see an enhancement in neuron-specific gene expression signatures in our sorted neuronal nuclei and our hiPSN model of cocaine exposure. The hiPSN model will help elucidate which effects are due to acute versus chronic exposure of cocaine. DISCUSSION/SIGNIFICANCE OF FINDINGS: Transcriptomic signatures found with this analysis can help us understand mechanisms of cocaine neurotoxicity in human neurons. With this work and future proposed studies, we can discover targetable molecular pathways to develop drugs that can reduce or reverse cocaine-related impairment.

